# Tricuspid annular displacement measured by 2-dimensional speckle tracking echocardiography for predicting right ventricular function in pulmonary hypertension: A new approach to evaluating right ventricle dysfunction

**DOI:** 10.1097/MD.0000000000011710

**Published:** 2018-07-27

**Authors:** Yidan Li, Yidan Wang, Yuanhua Yang, Mingxi Liu, Xiangli Meng, Yanping Shi, Weiwei Zhu, Xiuzhang Lu

**Affiliations:** a Department of Echocardiography, Heart Center; b Department of Respiratory and Critical Care Medicine; c Department of Radiology, Beijing Chao Yang Hospital, Capital Medical University, Beijing, China.

**Keywords:** pulmonary hypertension, right ventricular function, speckle tracking imaging, tricuspid annular longitudinal displacement (TMAD)

## Abstract

This study aimed to determine the ability of tricuspid annular displacement measured by 2-dimensional speckle tracking echocardiography (STE) to predict right ventricular (RV) dysfunction in pulmonary hypertension (PH) patients. Here, we present a new method for assessing RV function that also employs STE and is based on measurement of tricuspid annular displacement.

A total of 225 patients were divided into 2 groups according to the pulmonary artery systolic blood pressure (PASP), estimated by echocardiographic measurement of tricuspid regurgitation: group I (PASP ≥50 mm Hg) and group II (36 mm Hg ≤ PASP <50 mm Hg). The tricuspid annular plane systolic excursion (TAPSE), RV index of myocardial performance (RIMP), RV fractional area change (RVFAC), tissue Doppler-derived tricuspid lateral annular systolic velocity (s′), and the tricuspid annular longitudinal displacement (TMAD) parameters were measured. Thirty patients underwent cardiac magnetic resonance (CMR) examination, and right ventricular ejection fraction (RVEF) was calculated.

The conventional parameters as well as the TMAD parameters differed significantly between the 2 groups (all *P* < .01). Good correlation was observed between the TMAD parameters and CMR-derived RVEF (all *P* < .01). The TMAD parameters had moderate predictive value for predicting RV dysfunction in PH patients (all *P* < .01). From receiver operating characteristic curves, we determined the optimal cut-off values for TMAD parameters for detecting RV dysfunction with good sensitivity and specificity.

The TMAD parameters can predict the decline of RV function in patients with PH and thus provide new diagnostic indices for clinical management of these patients.

## Abstract 2

Supplemental Digital Content is available in the text

## 1 Introduction

Pulmonary hypertension (PH) is a pathophysiological disorder that may stem from multiple clinical conditions and presents as a complication of most cardiovascular and respiratory diseases.^[1]^ Right ventricular (RV) dysfunction in PH patients is associated with adverse outcomes, and thus, early detection of RV dysfunction has become increasingly important. Progressive right heart failure is the main cause of death among patients with PH.^[2]^ Therefore, careful assessment of right heart function in PH patients is critical, and echocardiography is the main approach used in such evaluations. Specifically, RV systolic function is assessed using multiple conventional parameters, including RV index of myocardial performance (RIMP), tricuspid annular plane systolic excursion (TAPSE), RV fractional area change (RVFAC), and tricuspid annular systolic velocity (s′), with TAPSE being the most commonly used. Each of these conventional parameters has limitations. TAPSE-M is a method for measuring the distance of systolic excursion of the RV annular segment along its longitudinal plane, but an obvious disadvantage of TAPSE is that it assumes the displacement of a single segment represents the function of a complex RV structure. Furthermore, it is angle dependent and may be load dependent. Many other methods and indicators have been proposed for evaluating right heart function, and tricuspid annular longitudinal displacement (TMAD) is an emerging modality based on speckle tracking echocardiography (STE) in which the annular tissue is tracked toward the RV apex for the purpose of evaluating ventricular function.^[3,4]^ Unlike RV wall motion analysis, TMAD measurement by STE offers the advantage of not being affected by endocardial definition and myocardial dropouts. Here, we present a new method for assessing RV function that also employs 2-dimensional (2D) STE and is based on measurement of tricuspid annular displacement. We compared 2D-STE measurements of TAPSE with RV functional parameters to investigate the clinical value of this approach for predicting altered RV function in patients with PH.

## 2 Patients and methods

### 2.1 Study patients

A total of 245 consecutive adult patients with definite (verified by right heart catheterization) or suspected pre-capillary PH (based on symptoms of chest tightness, shortness of breath, dyspnea, or history of venous thrombosis of lower extremities) presented between January 2014 and February 2016 at Beijing Chaoyang Hospital and were retrospectively studied. Fifteen cases with poor echocardiographic image quality, 2 cases with atrial fibrillation, 2 cases with moderate to severe mitral regurgitation, and 1 case with moderate aortic regurgitation were excluded. The mean patient age was 48.8 ± 15.3 years, and 186 patients (82.7%) were women. Patients were divided into 2 groups according to the pulmonary artery systolic blood pressure (PASP), estimated by echocardiographic measurement of tricuspid regurgitation: group I (PASP ≥50 mm Hg) and group II (36 mm Hg ≤ PASP < 50 mm Hg).^[5]^ Right atrial pressure can be estimated by echocardiography based on the diameter and respiratory variation in diameter of the inferior vena cava: an IVC diameter < 2.1 cm that collapses >50% with a sniff suggests a normal RA pressure of 3 mm Hg, whereas an IVC diameter >2.1 cm that collapses <50% with a sniff suggests a high RA pressure of 15 mm Hg.^[1]^ The study was conducted according to the guidelines of the Declaration of Helsinki and was approved by the Ethics Committee of Beijing Chaoyang Hospital. Written informed consent was obtained from all participants.

### 2.2 Echocardiographic examination

All 225 patients underwent echocardiographic examination to assess right heart function, following the recommendations of the Guidelines for the Echocardiographic Assessment of the Right Heart in Adults.^[6]^ Images were obtained with the patient in the left lateral decubitus position using either a Philips EPIQ 7C (Philips Healthcare, MA) or Philips IE33 (Philips Healthcare). The TAPSE was acquired by M-mode; the M-mode cursor was placed through the lateral aspect of the tricuspid annulus, such that the annulus moved along the M-mode cursor. The systolic displacement was measured from end-diastole to end-systole. The tricuspid s′ was measured via tissue Doppler imaging in the apical 4-chamber view. The isovolumic acceleration of the RV was calculated as the peak isovolumic myocardial velocity divided by the time to peak velocity, as measured by tissue Doppler imaging at the lateral tricuspid annulus. The RV end-diastolic area (RV EDA) and RV end-systolic area (RV ESA) were obtained from the 2-D apical 4-chamber view. The RVFAC was calculated as: RVFAC = (RV diastolic area – RV systolic area)/RV diastolic area × 100%. The RIMP was calculated as the ratio of the isovolumic time to the ejecting time, which was measured during the same pulsed tissue Doppler imaging. The isovolumic time was calculated by subtracting the ejecting time from the tricuspid closure time. The ratio of the RV transverse diameter to the left ventricular transverse diameter was measured at the base of end-diastole using the apical 4-chamber view. According to the updated 2015 ASE guidelines and standards for cardiac chamber quantification by echocardiography in adults, the criteria for RV dysfunction were TAPSE <17 mm, RIMP >0.54, FAC <35%, and s′<9.5 cm/s.^[7]^

### 2.3 2D-STE analysis

**Figure 1:**
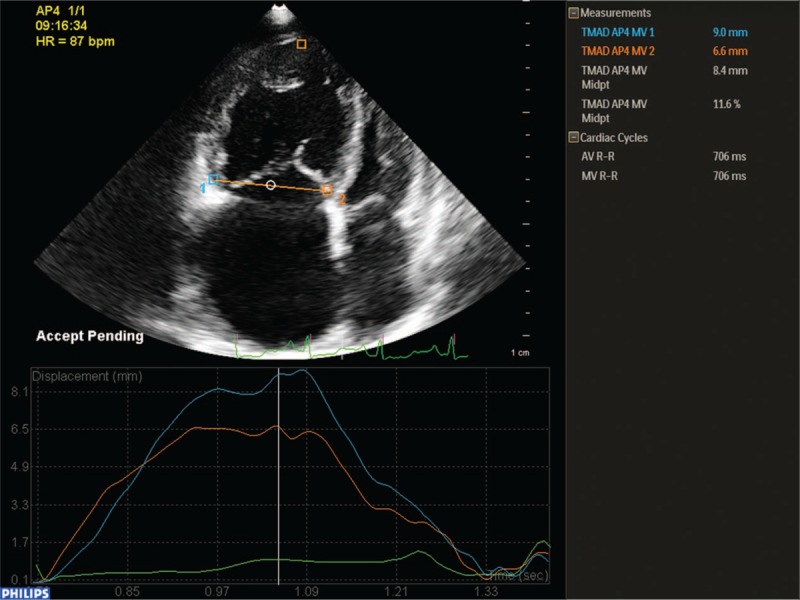
2D-STE measurement of TMAD parameters. Along the entire right ventricle and using the RV-focused view, user-defined anatomic landmarks point 1 (blue square) and point 2 (orange square) were placed at the bottom of the RV free wall and the bottom of the interventricular septum, respectively, and another orange square was placed at the apex of the right ventricle. Plots of TMAD1, TMAD2, TMADm, and TMADm% values are displayed.

Apical 4-chamber views were specifically optimized to visualize the right ventricle to obtain echocardiographic cine loops by recording 3 consecutive heart cycles (frame rates >61 frame/s), and the data were stored in a mobile device using the DICOM format. Offline analyses were performed using QLAB 10.3 software (Philips Healthcare). Three points were selected in the RV-focused apical 4-chamber view as user-defined anatomic landmarks: insertion of the anterior and septal leaflets into the tricuspid annulus and the RV apex. The software automatically tracked the TMAD and calculated the TMAD at the RV free wall (TMAD1), the TMAD at the interventricular septum (TMAD2), the TMAD at the midpoint of the tricuspid annulus (TMADm), and the RV longitudinal shortening (TMADm%) (Fig. 1). These parameters were measured 3 times, and averages were recorded in unity drawing form.

### 2.4 CMR

**Figure 2:**
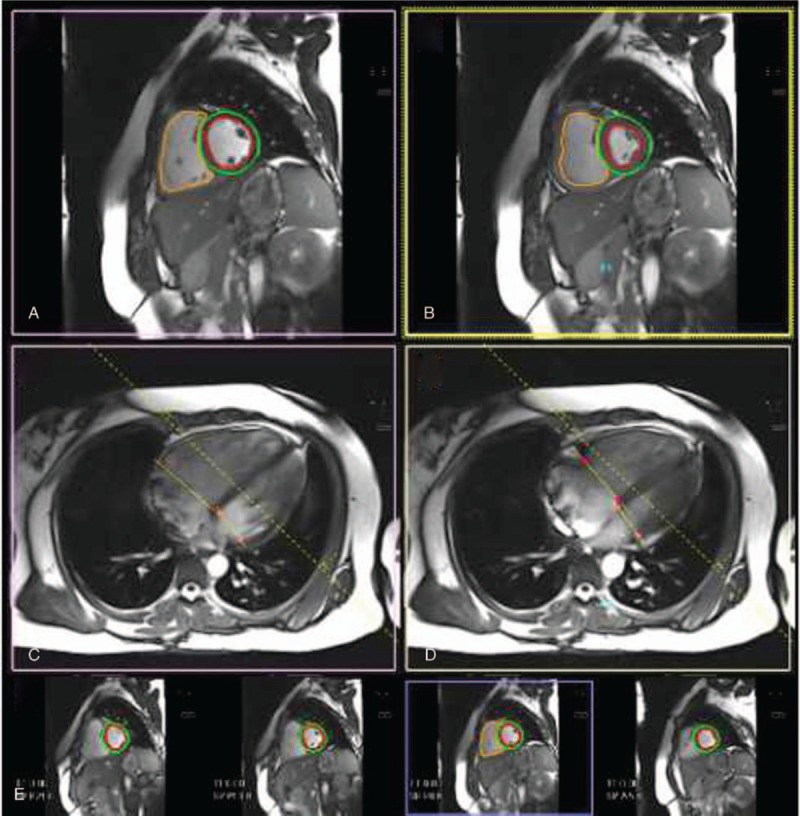
Right ventricular (RV) and left ventricular (LV) chamber quantification. For RV volume quantification, the endocardial (orange) contours are delineated in diastole (A and C) and systole (B and D) in a stack of short-axis slices (E) that cover the whole RV. For LV chamber quantification, the endocardial (red) and epicardial (green) contours are delineated in diastole (A and C) and systole (B and D) in a stack of short axis slices (E) that cover the whole left ventricle with inclusion of the papillary muscles as part of the LV volume. (A, B) Corresponding mid-short axis in diastole (A) and systole (B). (C, D) The 4-chamber images in diastole (A) and systole (B) covering the RV and LV that enable best identification of the tricuspid valve plane and mitral valve plane, respectively.

Cardiac magnetic resonance (CMR) imaging was performed in 30 patients using a 3.0-Tesla magnetic resonance scanner (TimTrio; Siemens, Erlangen, Germany) and a heart-liver coil for data acquisition. All CMR data were transferred to a workstation (Syngo, Via VE30A; Siemens, Berlin, Germany) and analyzed with validated software (cardiac analysis, ventricular function; Siemens Medical Systems, Erlangen, Germany). CMR was performed with the Cine sequence (true fast imaging with steady precession, True FISP; repetition time/echo time, 44.24/1.41 ms; flip angle, 50°; matrix, 192 × 192 pixels; field of view, 340 mm; section thickness, 6 mm) using retrospective electrocardiogram triggering during breath holding. Twenty-five frames were reconstructed for each cardiac cycle. The right and left ventricles were semi-automatically segmented by an experienced radiologist who identified the endocardial and epicardial boundaries. Function parameters, including the RV end-diastolic volume (RVEDV), RV end-systolic volume (RVESV), RV ejection fraction (RVEF), RV stroke volume, and RV cardiac output were automatically calculated by the software. Similar cardiovascular parameters were obtained for the left ventricle using the same methods (Fig. 2). The time interval between CMR and echocardiography was less than 72 hours.

### 2.5 Statistical analyses

Continuous variables are expressed as mean ± standard deviation. Normality was evaluated using the 1-sample Kolmogorov–Smirnov test. Comparisons between PH patients and controls were performed using a Student *t* test for continuous normally distributed variables, Mann–Whitney test for continuous non-normally distributed variables, and Fisher exact test for categorical variables. Linear regression analysis was used to study the relationships between 2 variables. The ability of TMAD to predict RV dysfunction was assessed using receiver operating characteristic (ROC) curves to calculate optimal values based on the area under the curve (AUC). ROC curves were constructed to identify the optimal cut-off value of TMADm% for detecting impaired RV function. The optimal cut-off value was defined as the point closest to 1 in the top left corner. ROC curves for the new echo markers were statistically compared with those of the conventional echo markers using the method of DeLong. *P* < .05 was considered statistically significant. SPSS statistical software (version 17.0 for Windows; SPSS, Inc., Chicago, IL) was used for statistical analysis and graphic presentation.

### 2.6 Reproducibility and reliability of the echocardiography measurements of TMAD parameters

The intraobserver agreement for measurements was assessed by comparing the measurements of repeated analysis in 20 randomly chosen patients (Yidan Wang). The interobserver agreement was assessed using the same patients (n = 20) by comparing the results measured by (Yidan Wang) and those obtained by a second, experienced cardiologist (Yidan Li). The second cardiologist was not aware of the echocardiography measurements of the first examiner. Reproducibility was assessed via Bland–Altman analysis and intraclass correlation coefficients (ICCs) between the 2 measurements.

## 3 Results

### 3.1 Patient characteristics

A total of 225 consecutive patients with definite or suspected PH were enrolled in this study. As summarized in Table 1, the study population included 39 men and 186 women with an average age of 48.8 ± 15.3 years. The cohort consisted of 89 patients with chronic thromboembolic PH or acute pulmonary embolism, 53 patients with idiopathic PH, 32 patients with pulmonary embolism, 32 patients with PH associated with connective tissue disease, 17 patients with PH associated with congenital heart disease, and 2 patients with heritable PH. One hundred eighty-two patients were assigned to the group I (PASP ≥50 mm Hg), and 43 patients met the criteria for inclusion in the group II (36 mm Hg ≤PASP <50 mm Hg). Groups I and II showed no significant differences in age, sex, body mass index (BMI), or other basic characteristics, whereas the left ventricular ejection fraction (LVEF), PASP, and area of tricuspid regurgitation (A_TR_) did differ significantly between the 2 groups (Table 1).

**Table 1:**
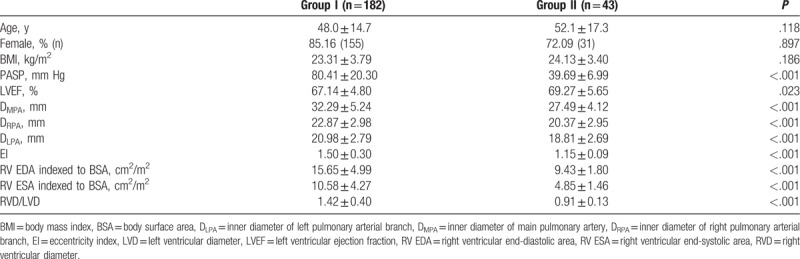
Patient characteristics.

### 3.2 Evaluation of RV structure and function

With regard to RV structure, significant differences were observed in the inner diameter of the main pulmonary artery (D_MPA_), inner diameter of the right pulmonary arterial branch DRPA (D_RPA_), inner diameter of the left arterial branch (D_LPA_), eccentricity index (EI), RV EDA, RV ESA, RV diameter (RVD), left ventricular diameter (LVD), and ratio of RVD/LVD between the group I and group II (Table 1). Overall, in the group I, the right heart was significantly enlarged, the increase in the size of the pulmonary artery was more obvious, and the EI was larger. Moreover, the RV load was significantly greater.

With regard to RV function, significant differences were observed in the conventional parameters of TAPSE, RIMP, RVFAC, and s′ between the 2 groups (Table 2). Only the ratio of the tissue Doppler-derived tricuspid annular early diastolic peak velocity (e′) to the tissue Doppler-derived tricuspid annular late diastolic peak velocity (a′) did not differ between the groups. In addition, all measured TMAD parameters (TMAD1, TMAD2, TMADm, and TMADm%) differed significantly between the groups (Table 2).

**Table 2:**
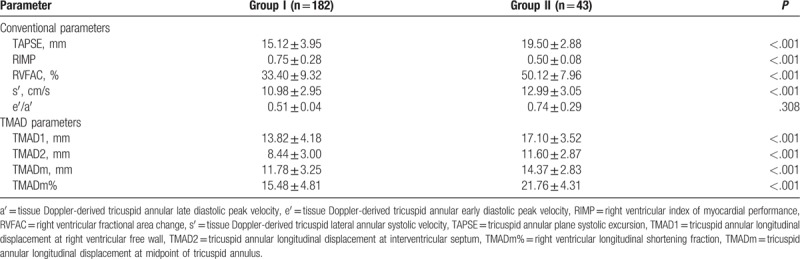
Conventional parameters of RV function and TMAD parameters.

### 3.3 Correlation analysis of echocardiographic RV function parameters and CMR-derived RVEF

According to the results shown in Supplementary Figure 1A to D, correlations were observed between the traditional echocardiographic RV function parameters (TAPSE, RVFAC, RIMP, and s′) and CMR-derived RVEF. The CMR-derived RVEF significantly correlated with RVFAC (*r* = 0.793; *P* < .001) and RIMP (*r* = -0.696, *P* < .001), but not with TAPSE (r = 0.411, *P* = .024) and s′ (*r* = 0.431, *P* = .017).

As shown in Supplementary Figure 2A-D, correlations were observed between the TMAD parameters (TMAD1, TMAD2, TMADm, and TMADm%) and CMR-derived RVEF. The CMR-derived RVEF significantly correlated with TMAD1 (*r* = 0.751; *P* < .001), TMAD2 (*r* = 0.606, *P* < .001), TMADm (*r* = 0.729, *P* < .001), and TMADm% (*r* = 0.797, *P* < .001).

### 3.4 Predictive value of TMAD parameters for RV dysfunction in patients with PH

The AUCs under the constructed ROC curves (Supplementary Figure 3A-D) indicated that TMAD1, TMAD2, TMADm, and TMADm% could all be used to predict RV dysfunction based on TAPSE, RIMP, RVFAC, and s′ measurements (all *P* < .01, Supplementary Tables 1–4). From the ROC curves, we determined the optimal cut-off values for each TMAD parameter for detecting TAPSE < 17 mm, RIMP >0.54, RVFAC < 35%, and s′ <9.5 cm/s with sensitivities and specificities (Supplementary Table 5).

As shown in Supplementary Figure 4A to D, the areas under the constructed ROC curves indicated that TMADm% could be used to predict RV dysfunction based on the CMR-derived RVEF as well as that RVFAC may be stronger than the other traditional parameters, although there was no statistically significant difference (Supplementary Table 6).

### 3.5 Reproducibility and reliability

Bland–Altman analysis of the intraobserver variability of echocardiography-derived TMAD parameters showed low mean differences and limits of agreement. Regarding the interobserver variability, Bland–Altman analysis showed similarly small mean differences and limits of agreement (Supplementary Figure 5A–D). ICCs were acceptably high for all measurements (Supplementary Table 7).

## 4 Discussion

RV function was highly sensitive to load change. Transthoracic echocardiography (TTE) is considered the mainstay screening modality for both PH and RV structure and function.^[8]^ Prediction of RV failure due to PH is difficult because RV failure develops rapidly in some patients and does not occur for years in others. Clinically, hypertrophy has been described as the initial adaptive response to PH, in order to increase contractility.^[9]^ As the condition progresses with sustained pressure overload, the increase in contractility is insufficient to maintain cardiac output,^[10,11]^ and eventually, RV failure characterized by increased filling pressure, diastolic dysfunction, and decreased cardiac output occurs. The relationship between the left ventricle and right ventricle can be quantitated by the EI, with an EI >1.0 suggesting RV overload. Indeed, in the present study, right heart overload was more obvious in group I than in group II, according to greater values for the RVD, pulmonary artery and branch diameter, and EI between the groups. Thus, the right ventricle is more than a passive chamber. In addition, a ratio of e’/a’ <0.52 is considered abnormal and indicative of RV diastolic dysfunction,^[7]^ and only the PH group in the present study showed RV diastolic dysfunction based on this value.

Studies have shown that longitudinal shortening of the myocardium is a greater contributor to RV stroke volume than circumferential shortening during normal contraction.^[12]^ In addition, regional myocardial shortening during contraction has been correlated with local transmural myocardial fiber orientations. Hill et al^[13]^ found that the density of combined myofibers and extracellular matrix remains relatively constant during progression from the normal to the hypertensive state, and the relative density of myofibers increases. Rain et al^[14]^ reported increases in the cardiomyocyte cross-sectional area and increased intrinsic stiffness in pulmonary artery hypertension (PAH) patients. Therefore, low TAPSE values are strongly correlated with RV dysfunction.^[15]^ We further hypothesized that in the later stages of RV failure; a loss of contractility in the longitudinally reoriented myofibers reduces longitudinal shortening and underscores the decrease in excursion measured with TAPSE.

Current clinical techniques that are used to diagnose and predict RV function include right heart catheterization and echocardiography-based measurement of TAPSE.^[16]^ One important advantage of these approaches over RV wall motion analysis is that these measurements are not affected by endocardial definition and myocardial dropouts. Knio et al^[17]^ used real-time 3D transesophageal echocardiography (TEE) to quantify the dimensions of the tricuspid annulus and analyze its geometric changes over the course of the cardiac cycle, and their analysis demonstrated that the tricuspid annulus is a nonplanar and dynamic structure. Malinowski et al^[18]^ found the tricuspid annulus is a saddle-shaped nonplanar structure with 2 horns in the anteroseptal and posterior regions. During acute PH and LV pressure overload, the TA dilates along all annular regions while the 3D annular geometry is essentially preserved. TAPSE predominantly reflects RV longitudinal function, and assuming that it always can represent the function of a complex structure is an oversimplification of its diagnostic performance.^[19]^ Our study demonstrated that the TMAD1 was higher than the TMAD2 and the values of TMAD parameters differed based on where the measurements were performed. Displacement was higher in the RV free wall than in the septal wall, and this is likely related to the existence of significant interventricular mechanical coupling via the septum, resulting in anchoring of the septal side of the tricuspid annulus, which is less pronounced at the free-wall side of the annulus. This finding is consistent with prior studies demonstrating that TAPSE is influenced by left ventricular function, likely due to ventricular interdependence.^[20]^

Assessment of RVEF is increasingly relevant in clinical practice, as recent echo and CMR studies have shown its prognostic importance. Although CMR is currently the gold standard for RVEF measurements, it is not feasible and practical in all patients. Our study revealed a good correlation between TMAD parameters and CMR-derived RVEF, especially TMADm%. Ahmad et al^[3]^ indicated that the STE-derived longitudinal shortening fraction provides an alternative method for fast, automated, reproducible, noninvasive, and quantitative evaluation of RV function. Our work explored the ability of the indices to correctly classify patients with RV dysfunction. From ROC curves, we determined optimal cut-off points for each parameter with good sensitivity and specificity. This methodology is likely to be of interest for the clinical management of patients with RV dysfunction. Alonso et al^[4]^ found good correlation between the speckle tracking tricuspid annulus parameters and RVEF calculated by CRM and explored the ability of the indices to correctly classify patients with reduced RVEF, obtaining an AUC values greater than 0.85 for all parameters.

In conclusion, TMAD values measured by 2D-STE have moderate value for predicting RV dysfunction in patients with PH. These parameters are simple and efficient to measure and provide a new approach for diagnosing and evaluating the severity of PH.

### 4.1 Study limitations

We acknowledge a few potential limitations in our study. First, the relatively small number of PH patients who obtained CMR examination was a major limitation. The majority of patients were women (82.7%), limiting the generalizability of the study. Second, the data for this group were not correlated with clinical prognostic indicators, such as brain natriuretic protein, 6-minute walk distance, and cardiac output. Thus, further studies are required to more thoroughly demonstrate the clinical value of the proposed parameters. The sensitivity and specificity values for TMAD metrics were calculated by applying the ROC cut-off values and need to be confirmed independently in a further prospective study.

## 5 Conclusion

Compared with TAPSE using a single segment to reflect the complex 3D structure of the right ventricle, TMAD parameters measured by 2D-STE offer a new approach to evaluating RV dysfunction in patients with PH. The TMAD parameters can predict RV dysfunction in patients with PH and thus provide new diagnostic indices for the clinical management of these patients.

## Acknowledgment

We would like to thank all patients who participated in the study.

## Author contributions

**Conceptualization:** Yidan Li, Yidan Wang, Yuanhua Yang, Yanping Shi, Xiuzhang Lu.

**Data curation:** Yidan Li, Yidan Wang, Yuanhua Yang, Mingxi Liu, Xiangli Meng, Yanping Shi, Weiwei Zhu, Xiuzhang Lu.

**Formal analysis:** Yidan Li, Yidan Wang, Yuanhua Yang, Mingxi Liu, Xiangli Meng, Yanping Shi, Weiwei Zhu, Xiuzhang Lu.

**Funding acquisition:** Yidan Li, Yidan Wang, Yuanhua Yang, Mingxi Liu, Xiangli Meng, Yanping Shi, Weiwei Zhu, Xiuzhang Lu.

**Investigation:** Yidan Li, Yidan Wang, Yuanhua Yang, Mingxi Liu, Xiangli Meng, Yanping Shi, Weiwei Zhu, Xiuzhang Lu.

**Methodology:** Yidan Li, Yidan Wang, Yuanhua Yang, Mingxi Liu, Xiangli Meng, Yanping Shi, Weiwei Zhu, Xiuzhang Lu.

**Project administration:** Yidan Li, Yidan Wang, Yuanhua Yang, Xiangli Meng, Yanping Shi, Xiuzhang Lu.

**Resources:** Yidan Li, Yidan Wang, Yuanhua Yang, Mingxi Liu, Xiangli Meng, Yanping Shi, Weiwei Zhu, Xiuzhang Lu.

**Software:** Yidan Li, Yidan Wang, Yuanhua Yang, Mingxi Liu, Xiangli Meng, Yanping Shi, Weiwei Zhu, Xiuzhang Lu.

**Supervision:** Yidan Li, Yidan Wang, Yuanhua Yang, Yanping Shi, Xiuzhang Lu.

**Validation:** Yidan Li, Yidan Wang, Yuanhua Yang, Mingxi Liu, Xiangli Meng, Yanping Shi, Xiuzhang Lu.

**Visualization:** Yidan Li, Yidan Wang, Yuanhua Yang, Mingxi Liu, Xiangli Meng, Yanping Shi, Weiwei Zhu, Xiuzhang Lu.

**Writing – original draft:** Yidan Li, Yidan Wang, Yuanhua Yang, Mingxi Liu.

**Writing – review & editing:** Yidan Li, Yidan Wang, Yuanhua Yang, Mingxi Liu, Xiangli Meng, Yanping Shi, Weiwei Zhu, Xiuzhang Lu.

## Supplementary Material

Supplemental Digital Content
