# Supplementary material for: Tricuspid annular displacement measured by 2-dimensional speckle tracking echocardiography for predicting right ventricular function in pulmonary hypertension: A new approach to evaluating right ventricle dysfunction
Source: Medicine (Baltimore). 2018 Jul 27;97(30):e11710. doi: 10.1097/MD.0000000000011710 (PMC6078723; doi:10.1097/MD.0000000000011710)
Supplement: Supplemental Digital Content [file medi-97-e11710-s001.doc]

**Tricuspid annular displacement measured by two-dimensional speckle tracking echocardiography for predicting right ventricular function in pulmonary hypertension**: **A novel approach to evaluating right ventricle dysfunction**

**Supplementary Figure Legends**

**Supplementary Figure 1.** Correlations between the conventional echocardiographic RV function parameters (TAPSE, RVFAC, RIMP, and s′) and CMR-derived RVEF. (A) Correlation between TAPSE and CMR-derived RVEF. (B) Correlation between RVFAC and CMR-derived RVEF. (C) Correlation between RIMP and CMR-derived RVEF. (D) Correlation between s′ and CMR-derived RVEF.

**Supplementary Figure 2.** Correlations between the TMAD parameters (TMAD1, TMAD2, TMADm, and TMADm%) and CMR-derived RVEF. (A) Correlations between TMAD1 and CMR-derived RVEF. (B) Correlations between TMAD2 and CMR-derived RVEF. (C) Correlations between TMADm and CMR-derived RVEF. (D) Correlations between TMADm% and CMR-derived RVEF.

**Supplementary Figure 3.** Receiver operating characteristic curves demonstrating the ability of the TMADm% against those of the traditional echocardiographic parameters (TAPSE, RVFAC, RIMP, and sʹ) to predict RV dysfunction according CMR-derived RVEF.

**Supplementary Figure 4.** Receiver operating characteristic curves demonstrating the ability of the TMAD parameters (TMAD1, TMAD2, TMADm, and TMADm%) to predict (A) TAPSE <17 mm, (B) RIMP >0.54, (C) FAC < 35%, and (D) s′ <9.5 cm/s.

**Supplementary Figure 5.** Reproducibility of the TMAD parameters (TMAD1, TMAD2, TMADm, and TMADm%). Inter- and intraobserver reliability of the TMAD parameters (TMAD1, TMAD2, TMADm, and TMADm%) were assessed with 20 subjects.

**Supplementary Table 1** Areas under the curve (AUCs) indicating the ability of TMAD1 to predict RV dysfunction according to different parameters in patients with PH

| Variable | AUC | Standard Error | *P* | 95% CI |
| --- | --- | --- | --- | --- |
| TAPSE | 0.819 | 0.028 | <0.001 | 0.764-0.873 |
| RIMP | 0.712 | 0.034 | <0.001 | 0.646-0.779 |
| RVFAC | 0.793 | 0.030 | <0.001 | 0.735-0.850 |
| s′ | 0.823 | 0.028 | <0.001 | 0.769-0.878 |

TMAD1, tricuspid annular longitudinal displacement at right ventricular free wall; TAPSE, tricuspid annular plane systolic excursion; RIMP, right ventricular index of myocardial performance; RVFAC, right ventricular fractional area change; s′, peak tricuspid annular systolic velocity; CI, confidence interval

**Supplementary Table 2** Areas under the curve (AUCs) indicating the ability of TMAD2 to predict RV dysfunction according to different parameters in patients with PH

| Variable | AUC | Standard Error | *P* | 95% CI |
| --- | --- | --- | --- | --- |
| TAPSE | 0.766 | 0.032 | <0.001 | 0.704-0.828 |
| RIMP | 0.746 | 0.034 | <0.001 | 0.679-0.813 |
| RVFAC | 0.773 | 0.032 | <0.001 | 0.710-0.836 |
| s′ | 0.811 | 0.030 | <0.001 | 0.753-0.869 |

TMAD2, tricuspid annular longitudinal displacement at interventricular septum; TAPSE, tricuspid annular plane systolic excursion; RIMP, right ventricular index of myocardial performance; RVFAC, right ventricular fractional area change; s′, peak tricuspid annular systolic velocity; CI, confidence interval

**Supplementary Table 3** Areas under the curve (AUCs) indicating the ability of TMADm to predict RV dysfunction according to different parameters in patients with PH

| Variable | AUC | Standard Error | *P* | 95% CI |
| --- | --- | --- | --- | --- |
| TAPSE | 0.818 | 0.028 | <0.001 | 0.762-0.873 |
| RIMP | 0.755 | 0.032 | <0.001 | 0.692-0.818 |
| RVFAC | 0.820 | 0.028 | <0.001 | 0.7365-0.875 |
| s′ | 0.874 | 0.023 | <0.001 | 0.828-0.919 |

TMADm, tricuspid annular longitudinal displacement at midpoint of tricuspid annulus; TAPSE, tricuspid annular plane systolic excursion; RIMP, right ventricular index of myocardial performance; RVFAC, right ventricular fractional area change; s′, peak tricuspid annular systolic velocity; CI, confidence interval

**Supplementary Table 4** Areas under the curve (AUCs) indicating the ability of TMADm% to predict RV dysfunction according to different parameters in patients with PH

| Variable | AUC | Standard Error | *P* | 95% CI |
| --- | --- | --- | --- | --- |
| TAPSE | 0.838 | 0.028 | <0.001 | 0.783-0.893 |
| RIMP | 0.722 | 0.037 | <0.001 | 0.649-0.794 |
| RVFAC | 0.833 | 0.030 | <0.001 | 0.775-0.892 |
| s′ | 0.829 | 0.028 | <0.001 | 0.774-0.883 |

TMADm%, right ventricular longitudinal shortening fraction; TAPSE, tricuspid annular plane systolic excursion; RIMP, right ventricular index of myocardial performance; RVFAC, right ventricular fractional area change; s′, peak tricuspid annular systolic velocity; CI, confidence interval

**Supplementary Table 5** Optimal cut-off values for each TMAD parameter for detecting RV dysfunction with corresponding sensitivity and specificity values

| Parameter | Optimal cut-off value (%) |  | TAPSE  <17 mm | RIMP  >0.54 | RVFAC <35% | s′  <9.5 cm/s |
| --- | --- | --- | --- | --- | --- | --- |
| TMAD1 | 14.05 | SEN (%) | 80.2 | 78.7 | 72.1 | 64.2 |
|  |  | SPE (%) | 70.9 | 64.7 | 76.7 | 84.8 |
| TMAD2 | 7.90 | SEN (%) | 81.3 | 86.7 | 78.7 | 73 |
|  |  | SPE (%) | 50 | 49.3 | 56.3 | 62.1 |
| TMADm | 10.85 | SEN (%) | 87.9 | 84 | 82 | 74.2 |
|  |  | SPE (%) | 61.2 | 54 | 68.9 | 78.8 |
| TMADm% | 16.05 | SEN (%) | 85.7 | 82.7 | 81.1 | 68.6 |
|  |  | SPE (%) | 67.9 | 60.7 | 78.6 | 81.8 |

SEN, sensitivity; SPE, specificity; TMAD1, tricuspid annular longitudinal displacement at right ventricular free wall; TMAD2, tricuspid annular longitudinal displacement at interventricular septum; TMADm, tricuspid annular longitudinal displacement at midpoint of tricuspid annulus; TMADm%, right ventricular longitudinal shortening fraction; TAPSE, tricuspid annular plane systolic excursion; RIMP, right ventricular index of myocardial performance; RVFAC, right ventricular fractional area change; s′, tissue Doppler–derived tricuspid lateral annular systolic velocity

**Supplementary Table 6** Areas under the curve (AUCs) indicating the ability of TMADm% and traditional echocardiographic parameters to predict RV dysfunction based on the CMR-derived RVEF

| Variable | AUC | Standard Error | 95% CI |
| --- | --- | --- | --- |
| TMADm% | 0.975 | 0.030 | 0.841-1.000 |
| TAPSE | 0.815 | 0.112 | 0.631-0.932 |
| RIMP | 0.833 | 0.114 | 0.653-0.944 |
| RVFAC | 0.963 | 0.035 | 0.822-0.999 |
| s′ | 0.889 | 0.086 | 0.720-0.974 |

all P>0.05. TMADm%, right ventricular longitudinal shortening fraction; TAPSE, tricuspid annular plane systolic excursion; RIMP, right ventricular index of myocardial performance; RVFAC, right ventricular fractional area change; s′, peak tricuspid annular systolic velocity; CI, confidence interval

**Supplementary Table 7 Variation of inter- and intraobserver measurements**

| Parameters | | Absolute mean difference (mean±SD) | Interclass correlation | 95% CI | | *P* |
| --- | --- | --- | --- | --- | --- | --- |
|  |  |  |  | Lower Upper | |  |
| TMAD1 | Inter- | 0.18±0.87 | 0.980 | 0.951 | 0.992 | <0.001 |
|  | Intra- | 0.40±1.11 | 0.970 | 0.961 | 0.994 | <0.001 |
| TMAD2 | Inter- | 0.37±0.79 | 0.965 | 0.914 | 0.986 | <0.001 |
|  | Intra- | -0.08±1.04 | 0.942 | 0.859 | 0.976 | <0.001 |
| TMADm | Inter- | -0.16±0.93 | 0.966 | 0.916 | 0.986 | <0.001 |
|  | Intra- | -0.04±0.72 | 0.980 | 0.950 | 0.992 | <0.001 |
| TMADm% | Inter- | -0.05±0.96 | 0.969 | 0.924 | 0.988 | <0.001 |
|  | Intra- | -0.10±0.79 | 0.980 | 0.974 | 0.996 | <0.001 |


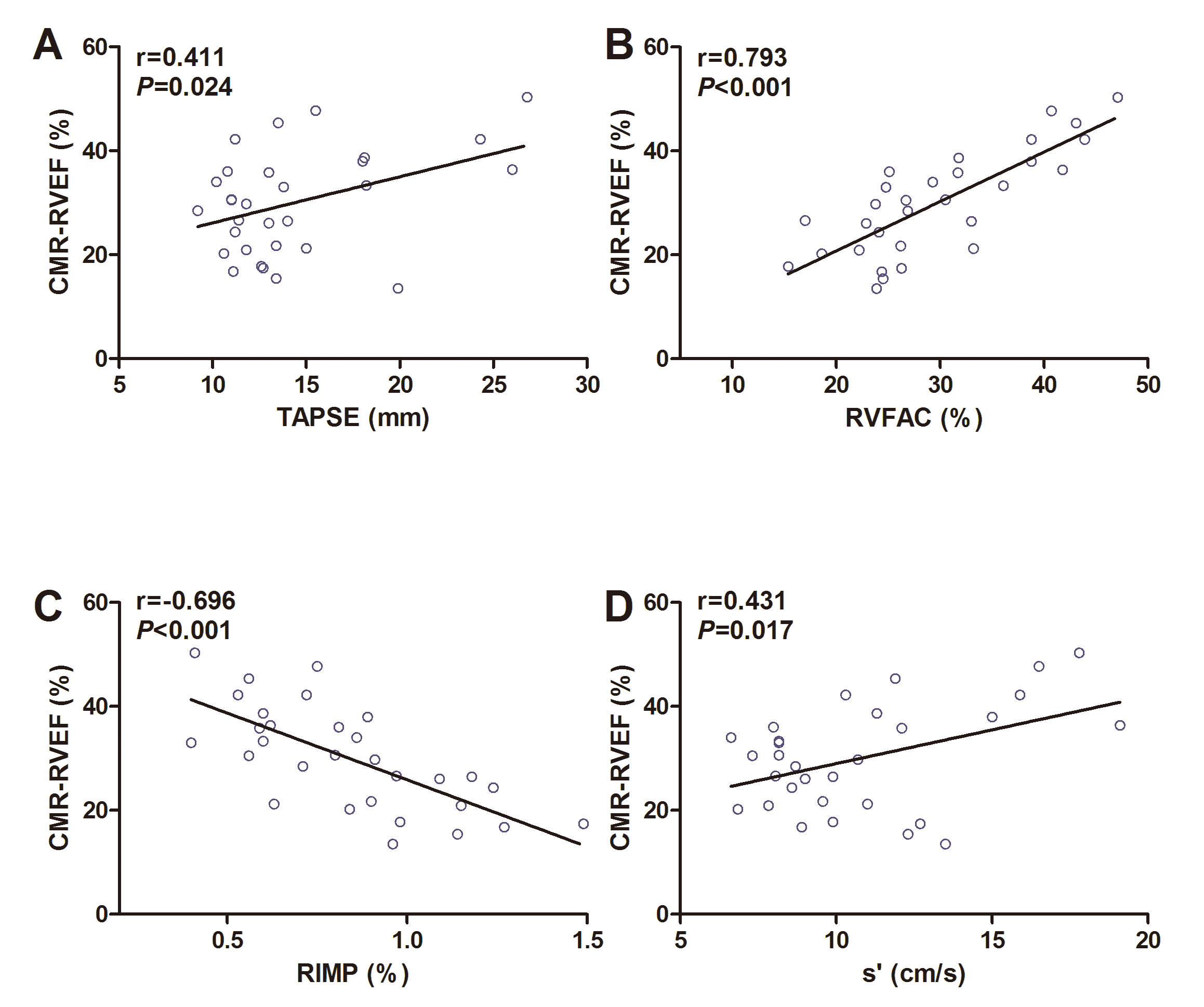


**Supplementary Figure 1**


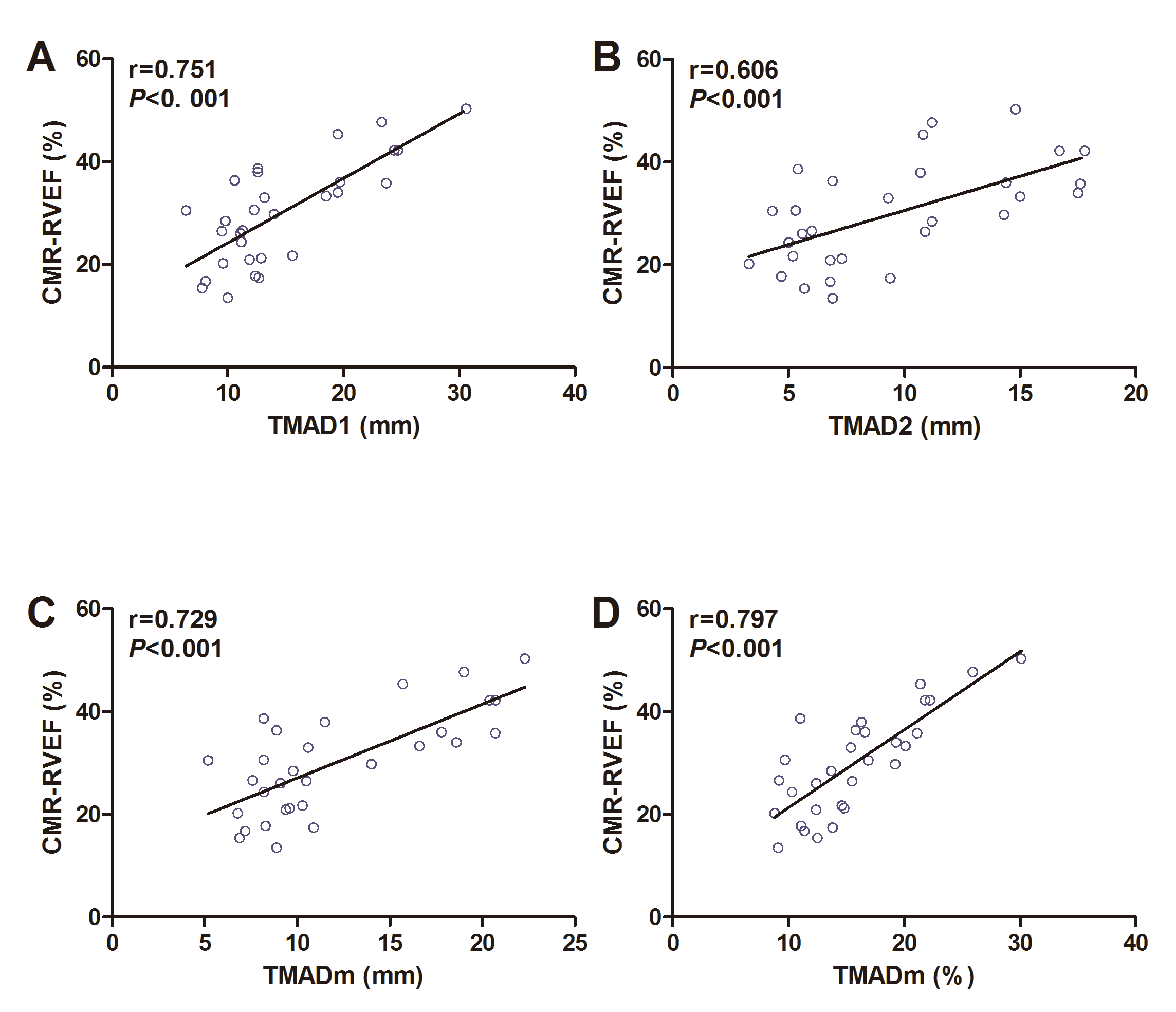


**Supplementary Figure 2**


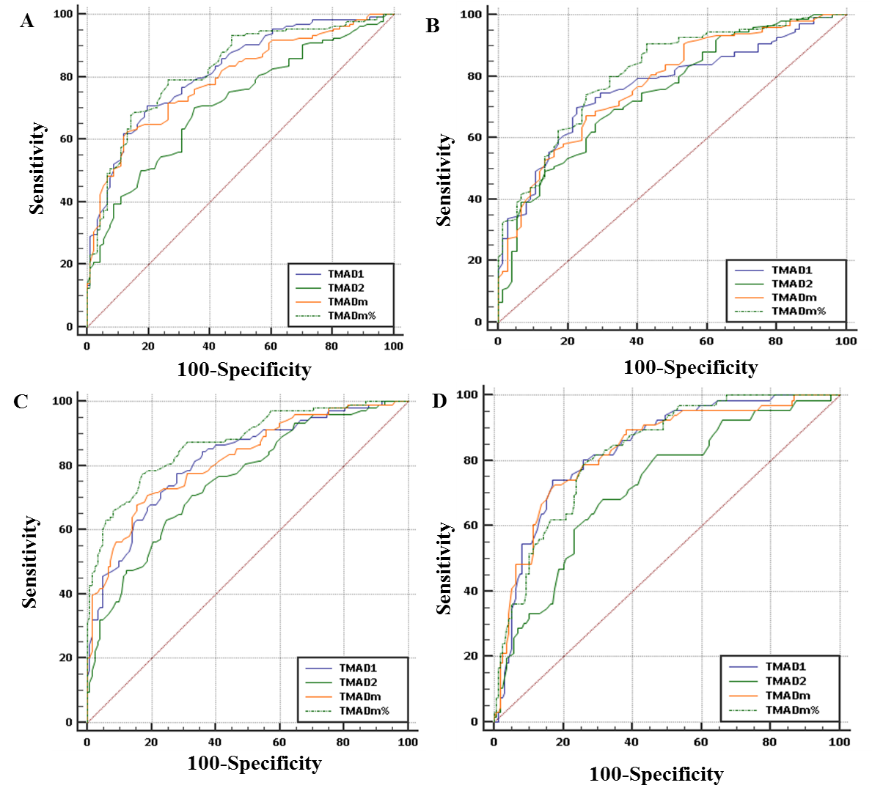


**Supplementary Figure 3**


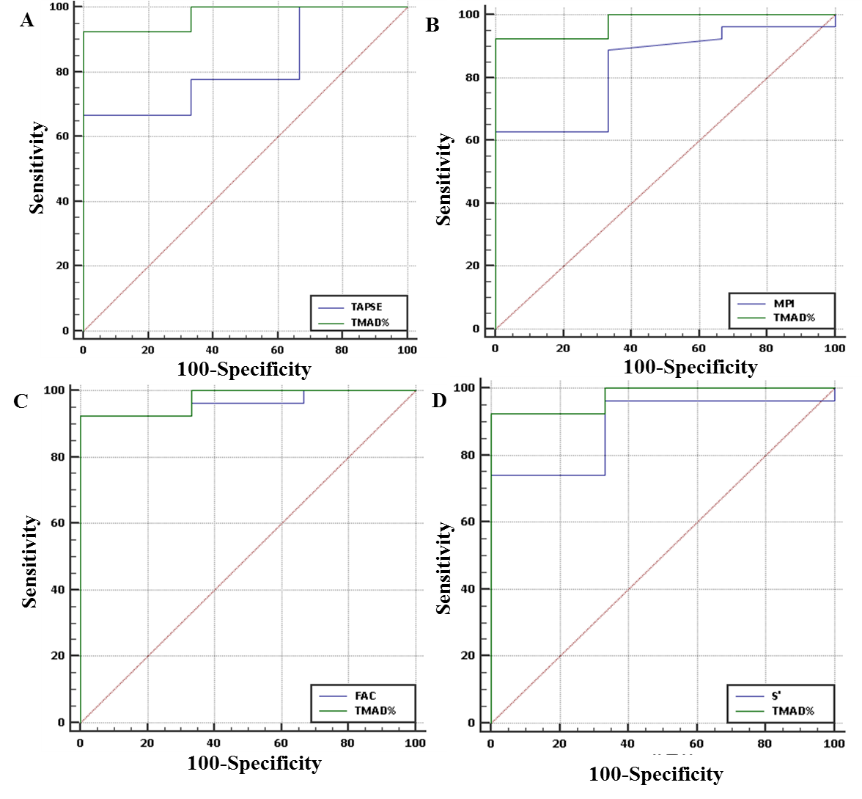


**Supplementary Figure 4**


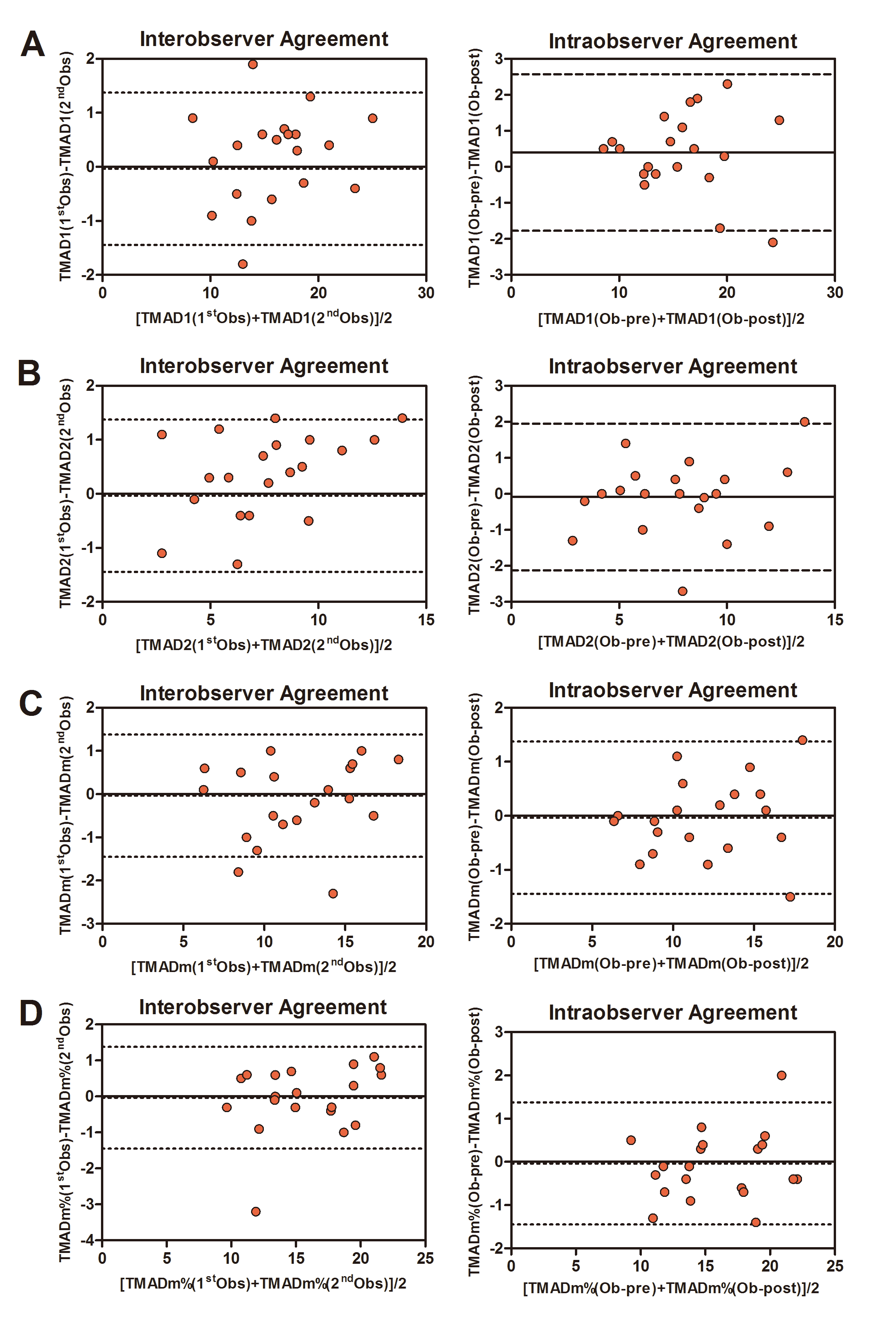


**Supplementary Figure 5**
